# A Modified BCA Method for Determination of Residual Protein in Enzymatic Biosynthetic Rebaudioside M

**DOI:** 10.1155/ijfo/2967051

**Published:** 2026-04-30

**Authors:** Feng Li, Wenjing Zhang, Jiaxi Bai, Xinjing Yang, Yanyan Huo, Yingjuan Qu

**Affiliations:** ^1^ School of Chemical Engineering, Xi’an University, Xi’an, 710065, Shaanxi Province, China, xjtu.edu.cn; ^2^ Xi’an Key Laboratory of Food Safety Testing and Risk Assessment, Xi’an, 710065, Shaanxi Province, China

**Keywords:** BCA method, enzymatic method, protein residues, quality control, Rebaudioside M

## Abstract

The sweetener Rebaudioside M (Reb M) has broad application prospects and can be produced on a large scale by enzymatic methods. However, detecting exogenous protein residues in Reb M products remains a major challenge, and methods for detecting protein residues in Reb M have rarely been reported. In this study, Reb M was dissolved in a sodium hydroxide solution to improve its solubility, and proteins were precipitated using deoxycholic acid sodium and trichloroacetic acid solutions. This precipitation method also eliminated the interference of Reb M in the detection results. After reconstituting the residual protein with water, the protein content in Reb M was measured using the bicinchoninic acid (BCA) method. The analytical method was internally validated according to the following parameters: range of linearity, limits of detection, limits of quantitation, sensitivity, accuracy, and precision. The optimized method was then successfully applied to the determination of protein residues in Reb A, Reb D, and Reb M. The recoveries ranged from 90.60% to 109.40%, and the intraday and interday relative standard deviation (RSD) for precision was both less than 5%. Results from 10 samples, including process products, final products, and products from different manufacturers, demonstrated that the method was suitable for the quantitative detection of residual proteins in enzyme‐catalyzed Reb M, providing technical support for the quality control of Reb M.

## 1. Introduction

In view of the escalating global prevalence of obesity and diabetes, the World Health Organization (WHO) recommends restricting sugar intake to less than 10% of total daily caloric intake, with a further reduction to less than 5% advised for additional health benefits [1–3]. As a result, the demand for low‐sugar and low‐calorie products that use sweeteners is steadily increasing. Sweeteners are significantly sweeter than sugar, with some being several hundred times sweeter than sucrose [4, 5]. Therefore, only a small amount is needed to achieve the desired sweetness, allowing for a substantial reduction in sugar use, which in turn helps control both calorie intake and blood sugar levels. However, conventional synthetic options, such as aspartame, acesulfame‐K, sucralose, and neotame, have raised health concerns regarding chronic consumption. In 2023, the WHO classified aspartame as a potential carcinogen, intensifying global scrutiny over the food safety of sweeteners. Natural sweeteners, particularly the zero‐calorie steviol glycosides (SGs) extracted from the leaves of *Stevia rebaudiana Bertoni*, represent the “third natural sweetening resource” after sugarcane and sugar beet [6]. With centuries of traditional use in South America, these compounds exhibit exceptional sweetening potency, negligible caloric content, and favorable sensory characteristics. Notably, they demonstrate anti‐inflammatory and antimicrobial properties, and their nonmetabolizable nature renders them biologically inert in humans, making them ideal sugar substitutes [7–9].

SGs, classified as diterpenoid glycosides with multiple sugar chains at the C13 and C19 positions of ent‐kaurene, comprise more than 40 identified compounds, including stevioside (ST), Rebaudioside A (Reb A), Rebaudioside B (Reb B), Rebaudioside D (Reb D), and Rebaudioside M (Reb M) [10]. Among them, Reb M is considered a next‐generation sweetener due to its high‐intensity sweetness, up to 350 times that of sucrose and its significantly reduced bitter aftertaste compared to ST and Reb A. However, the content of Reb M in *Stevia rebaudiana Bertoni* leaves is relatively low (0.4%–0.5% of leaf dry weight), in contrast to ST (5%–10%) and Reb A (2%–4%). This low natural abundance hinders its large‐scale production through traditional phytoextraction methods for commercial use [11–14]. Consequently, the search for efficient and cost‐effective synthesis methods has become a key research focus. The primary synthetic approaches for Reb M currently include chemical synthesis, fermentation, and enzymatic methods [15, 16]. The elucidation of Reb M’s enzymatic biosynthetic pathways has positioned biocatalytic synthesis as the predominant manufacturing paradigm, owing to its environmental sustainability, mild reaction conditions, operational simplicity, and enhanced yield efficiency [17, 18]. Industry leaders, such as Tate & Lyle (FDA GRAS No. 780) and INGIA‐BIO (FDA GRAS No. 799), utilize sequential enzymatic modification processes to convert the precursor Reb A to Reb M via the intermediate Reb D through the coordinated action of UDP‐dependent glucosyltransferases (UGTs) and sucrose synthases.

Notably, the incorporation of exogenous enzymatic proteins necessitates rigorous quality control, as residual proteins pose regulatory compliance challenges. Despite continuous improvements in enzymatic processes and purification technologies, proteins from the enzymatic reactions may still remain in the final products, potentially causing adverse reactions in humans. Therefore, controlling residual proteins in enzyme‐catalyzed products is a critical aspect of process control in the production of fermentation‐derived SGs [19]. While EU regulations mandate a protein residue limit of 5 mg/kg in SG Reb A, the FDA GRAS submissions by Tate & Lyle and INGIA‐BIO report residual protein levels of no more than 5 ppm in Reb M products, as determined by the bicinchoninic acid (BCA) assay method, although essential analytical validation data remain undisclosed [20, 21]. Regulatory frameworks also differ across regions: The WHO has not issued specific guidance on Reb M protein residues, and China’s GB1886.355–2022 standard for stevia extracts omits any specification for protein residues, highlighting the evolving nature of global food safety governance [24]. In summary, institutions and companies have not provided specific data and testing processes for protein residue detection, which makes it unknown whether their prescribed limits and methods have been fully validated.

Current Reb M documentation reveals inconsistencies in the units used to report protein residues, with both ppm and mg/kg cited interchangeably leading to potential analytical discrepancies. Most FDA GRAS submissions ambiguously report protein residues as ppm without clarifying the measurement basis. An exception is Manus Bio (FDA GRAS No. 1010), which explicitly quantifies protein content on a w/v basis via the BCA assay. The detection limit of their method is 22.5 ppm (22.5 mg/L) at a Reb M solution concentration of 1 g/L that translates to 22,500 mg/kg under w/w parameters, exposing significant challenges in unit conversion and cross‐study comparison. This terminological ambiguity underscores the need for standardized metrological protocols in regulatory documentation. Current methodologies for analyzing residual proteins in Reb M also vary widely. For example, PureCircle’s GRAS (No. 745) utilizes SDS‐PAGE analysis with a detection threshold of 10,000 ppm. It is three orders of magnitude higher than Manus Bio’s 22.5 ppm BCA‐based protocol. This stark contrast exists alongside regulatory asymmetries: The EU mandates less than 5 mg/kg, while Tate & Lyle and INGIA‐BIO claim compliance with no more than ppm without clear unit harmonization [22]. The increasing domestic demand for high‐purity Reb M and the emergence of new enzymatic Reb M products in regulatory pipelines expose systemic quality control vulnerabilities rooted in unstandardized quantification frameworks. To ensure product integrity and consumer safety, regulatory modernization must mandate the following. It should have explicit specification of protein residue limits in pharmacopeial standards, standardized unit declarations (*w*/*w* *v* *s*. *w*/*v*), and analytical method validation with sensitivity aligned to the claimed detection limits. Such harmonization is imperative to reconcile industrial practices with evolving food safety paradigms. In addition, solubility is crucial for the detection of protein residues. The solubility of Reb D and Reb M in water at room temperature is 0.5 mg/kg and 1.5 mg/kg, respectively, while the solubility of Reb A in water is 278.2 mg/mL. The higher solubility results in lower detection limit. This may be due to the high solubility of Reb A, which led the European Union to limit the detection of protein residues in the entire stevia series to 5 mg/kg, without considering the very poor water solubility of Reb D and Reb M.

At present, protein analysis technologies are advancing rapidly, and several methods are commonly used for protein detection [23, 24]. These include the SDS‐PAGE, the Lowry method, the Bradford method, and the BCA method. SDS‐PAGE is a classic and widely accepted standard in protein analysis, particularly effective for studying large molecular substances. However, it is often time‐consuming and labor‐intensive. The Lowry and Bradford methods, while frequently employed, suffer from limitations, such as interference from reagents and relatively low sensitivity. In contrast, the BCA method is widely used for quantifying soluble proteins in various matrices, including milk, plasma, tears, plant and animal tissues, food products, and urine. This method is based on the formation of a coordinated complex between Cu(II) ions and the nitrogen atoms in the peptide amide bonds, resulting in a color change from blue to violet. However, certain sample components, such as detergents, 2‐mercaptoethanol, carbohydrates, ammonium, flavonoids, hemoglobin, and salts, can interfere with the reaction, leading to either overestimation or underestimation of protein content. Therefore, selecting an appropriate detection method is essential to minimize interference and obtain reliable results [25, 26]. In the case of Reb M, its inherent hydrophobicity and reactivity with assay reagents can cause spectral interference, systematically inflating absorbance values beyond actual protein content. To address this analytical challenge, Fan et al. introduced a pretreatment protocol involving sodium deoxycholate‐mediated protein stabilization followed by trichloroacetic acid precipitation [27]. This approach effectively isolates proteins from matrix interference before BCA quantification.

The aim of the present work was to develop an accurate BCA‐based method for determining residual protein in Reb M [28]. The units of protein residue were standardized as mg/kg (w/w). In addition, we optimized the pretreatment protocol, selected appropriate solvents, and improved the solubility of Reb M. The finalized method was evaluated for its range of linearity, limits of detection (LODs), limits of quantitation (LOQs), sensitivity, accuracy, and precision. This validated workflow enables precise monitoring of residual protein levels throughout the manufacturing process, supporting both quality control standardization and purification process optimization. It is worth mentioning that the selection of appropriate Reb M solvent effectively solves the problem of Reb M being difficult to dissolve in water. Reb M dissolution is a prerequisite for detecting protein residues in Reb M, and Reb M has strong matrix interference on detection, requiring two cycles of trichloroacetic acid precipitation protein treatment. We also found that the treatment method of repeatedly adding trichloroacetic acid to precipitate protein did not affect protein quantification at all. Importantly, this improved BCA method for detecting protein residues was not mentioned in previous literature. The optimized method was then successfully applied to the determination of protein residues in Reb A, Reb D, and Reb M. This systematic approach helps establish critical quality attributes for enzymatic Reb M production and informs the design of rational downstream processing strategies.

## 2. Materials and Methods

### 2.1. Samples and Reagents

Bovine serum albumin (BSA) (Solarbio Science & Technology Co., Ltd., Beijing; Batch 1229Z051, 98% purity); 2,2′‐biquinoline‐4,4′‐dicarboxylic acid disodium salt (Sigma‐Vetec); anhydrous sodium carbonate (Macklin Biochemical Co., Ltd., Shanghai); sodium tartrate (Sinopharm Chemical Reagent Co., Ltd.); sodium hydroxide (Sinopharm Chemical Reagent Co., Ltd.); sodium bicarbonate (Titan Scientific Co., Ltd., Shanghai); copper (II) sulfate pentahydrate (Sinopharm Chemical Reagent Co., Ltd.); sodium deoxycholate (Macklin Biochemical Co., Ltd., Shanghai); and trichloroacetic acid (Titan Scientific Co., Ltd., Shanghai). All chemicals were of analytical grade. Experimental procedures utilized ultrapure water (18.2 MΩ cm resistivity) generated by a Milli‐Q water purification system (Millipore Corporation, USA).

Reb M test samples were obtained from a biotechnology enterprise and included the following batch identifiers: Reb M‐Process 1, Reb M‐Process 2, Reb M‐2301, Reb M‐2302, and Reb M‐2303.

Commercially available products of Reb M were also sourced from five different manufacturers, with batch numbers: 20230327, 20240902, 02BRMGA0122014, Z046RP2306, and 240709006. These manufacturers have distributed their products in the European Union, the United States, and China. The Reb A and Reb D validation samples are from a biotechnology company in Shanghai, with batch numbers RA‐230506 and RD‐230701.

### 2.2. Equipment and Instrument

Ultraviolet–visible (UV–Vis) spectrophotometer (Model UV‐1900i, Shimadzu Corporation [China] Management Co., Ltd.); analytical balance (Model SQP; Sartorius Scientific Instruments Co., Ltd.); and compact high‐speed centrifuge (Model 5425R; Eppendorf AG, Germany).

### 2.3. Preparation of Standard Solutions

BCA Reagent A: Precisely weigh 1 g of 2,2′‐biquinoline‐4,4′‐dicarboxylic acid disodium salt, 2 g anhydrous sodium carbonate, 0.16 g sodium tartrate, 0.4 g sodium hydroxide, and 0.95 g sodium bicarbonate. Dissolve in ultrapure water and adjust to a final volume of 100 mL. The resulting solution exhibits a pH of 11.2 ± 0.1.

BCA Reagent B: Accurately weigh an appropriate quantity of copper (II) sulfate pentahydrate and dissolve in ultrapure water to prepare a 4% (w/v) aqueous copper sulfate solution.

BCA Reagent C: Mix BCA Reagent A and BCA Reagent B at a volumetric ratio of 50:1. This working solution must be freshly prepared before use.

Sodium Deoxycholate Test Solution: Dissolve sodium deoxycholate in ultrapure water to obtain an aqueous solution containing 15 mg per 10 mL.

Trichloroacetic Acid Test Solution: Prepare an aqueous solution by dissolving 7.2 g of trichloroacetic acid in 10 mL of ultrapure water.

Sodium Hydroxide Solution: Dissolve 4 g of sodium hydroxide in 100 mL of ultrapure water to prepare a 4% (w/v) aqueous solution.

Bovine Serum Albumin (BSA) Reference Stock Solution: Prepare a 1.0 mg/mL aqueous solution of BSA.

BSA Reference Working Solutions: Dilute aliquots of the BSA reference stock solution with the sodium hydroxide solution to obtain calibration standards at concentrations of 2, 2.5, 5, 7.5, and 10 mg/L.

Reb A Sample Solution: Accurately weigh an appropriate amount of Reb M, dissolve it in the sodium hydroxide solution, and dilute to a final concentration of 120 mg/mL. Prepare three independent replicates.

Reb A Spiked Sample Solutions: Dissolve Reb M in the sodium hydroxide solution, then add defined volumes of the BSA reference stock solution to prepare mixtures containing 120 mg/mL Reb M and 2, 5, or 10 mg/L BSA.

Reb D Sample Solution: Accurately weigh an appropriate amount of Reb M, dissolve it in the sodium hydroxide solution, and dilute to a final concentration of 50 mg/mL. Prepare three independent replicates.

Reb D Spiked Sample Solutions: Dissolve Reb M in the sodium hydroxide solution, then add defined volumes of the BSA reference stock solution to prepare mixtures containing 50 mg/mL Reb M and 2, 5, or 10 mg/L BSA.

Reb M Sample Solution: Accurately weigh an appropriate amount of Reb M, dissolve it in the sodium hydroxide solution, and dilute to a final concentration of 50 mg/mL. Prepare three independent replicates.

Reb M Spiked Sample Solutions: Dissolve Reb M in the sodium hydroxide solution, then add defined volumes of the BSA reference stock solution to prepare mixtures containing 50 mg/mL Reb M and 2, 5, or 10 mg/L BSA.

Blank Solution: Sodium hydroxide solution prepared as described above.

### 2.4. Sample Preparation

Interference Removal Protocol: To 1.0 mL of the test solution, add 0.1 mL of the sodium deoxycholate test solution. Vortex thoroughly and incubate at 25°C for 10 min. Then, add 0.3 mL of the trichloroacetic acid test solution, vortex again, and centrifuge at 12,000 rpm for 15 min. Carefully discard the supernatant. Resuspend the resulting protein pellet in 1.0 mL of sodium hydroxide solution, and repeat the vortexing, incubation, and centrifugation steps as described above. Reconstitute the final protein pellet with BCA Reagent C and incubate the mixture at 37°C for 30 min. After incubation, allow the solution to cool to ambient temperature. Measure absorbance at 562 nm using a spectrophotometer. To ensure uniform reaction timing across all test samples and reference standards, implement a stopwatch‐controlled protocol with 60‐second intervals between sequential reagent additions to each test tube.

### 2.5. Optimization of Solvents, Reb M Concentration, and Pretreatment Cycles

The solubility of Reb M was evaluated in various solvents including H_2_O, 60% DMSO, 60% ethanol, and 1 M NaOH, and BSA recovery at a spiked concentration of 20 mg/L. The Reb M concentration was evaluated from 20 to 60 mg/mL using the protocol outlined in Section 2.4. Meanwhile, the Reb M absorbance of processing cycles was compared between untreated, first pretreatment, and second pretreatment.

### 2.6. Method Validation Study

The method performance was assessed by evaluating the accuracy, precision, linearity, LOD, and LOQ. The accuracy was expressed in terms of recovery, which was determined by spiking blank samples in three replicates at three concentration levels (40, 100, and 200 mg/kg). The precision was represented by the repeatability relative standard deviation (RSD) values on the same day. The linearity of the method was evaluated using the calibrated absorbance at a concentration ranging from 20 to 100 mg/L. Finally, the LODs and LOQs were calculated. To calculate the standard deviation (SD), the absorbance of the blank solution was measured, and the SD was derived from the response values and the slope of the standard curve. The LODs and LOQs were calculated as the concentration corresponding to the level of the 3.3·SD/*k* and 10·SD/*k*, respectively.

## 3. Results and Discussion

### 3.1. Method Optimization

The choice of dissolution conditions was mainly associated with the physical and chemical properties of the targeted compounds, as well as the properties of the solvent. In this study, the solubility conditions were a necessary prerequisite for the satisfactory performance of the method, given its extensive applicability in protein residue analysis. The solubility of Reb A, Reb D, and Reb M in water is 278.2 mg/mL, 0.4 mg/mL, and 1.5 mg/mL, respectively. Therefore, we first evaluated the solubility of Reb M in four solvents including pure H_2_O, 60% DMSO, 60% ethanol, and 1 M NaOH. The experimental results indicated that Reb M exhibits limited solubility in pure H_2_O with less than 5 mg/mL. The solubility in pure H_2_O is too poor to conduct further experiments. However, the solubility was much improved reaching 20 mg/mL in 60% ethanol (v/v) and 60% DMSO (v/v). Significantly, solubility in 1 M NaOH reached up to 60 mg/mL. Further, more recoveries of target protein BSA fell into the acceptable interval of 90%–110% in 60% DMSO, 60% ethanol, and 1 M NaOH, respectively, at a spiked concentration of 20 mg/L Reb M as summarized in Table 1 to assess the impact of solvent choice on protein detection results, with 1 M NaOH demonstrating superior performance.

**TABLE 1:** Protein recovery from Reb M spiking with different solvents and Reb M concentration.

Solvent	Reb M /(mg/mL)	Spiked BSA amount /(mg/L)	Measured BSA amount /(mg/L)	Recovery rate /%
60% DMSO	20	20	17.58	87.9
60% Ethanol	20	20	18.07	90.3
1 M·NaOH	20	20	18.63	93.2
1 M·NaOH	50	20	18.90	94.5
1 M·NaOH	60	20	18.04	90.2

Given a constant protein concentration, increasing the Reb M concentration improved detection sensitivity. Accordingly, the Reb M concentration was raised from 20 to 50 mg/mL, reducing the relative protein concentration (20 mg/L BSA) from 0.10% (1000 mg/kg) to 0.04% (400 mg/kg), while still maintaining high recoveries of 93.2% and 94.5%. However, further increasing the Reb M concentration to 60 mg/mL led to increased solution viscosity, which impaired protein sedimentation during centrifugation, resulting in supernatant contamination and a reduced recovery rate (90.2%). Further investigation was conducted on the effect of different NaOH concentrations on protein recovery. About 0.5 M, 1 M, and 2 M NaOH solutions were used as Reb M solvents, and 20 mg/mL protein was added to verify the recovery effect. Table 2 shows that the recovery rates of protein in 0.5 M and 1 M NaOH solutions were 88.70% and 98.25%, respectively, while there was no recovery in 2 M NaOH solution. The reason for this is that 2 M NaOH is too alkaline, and the TCA added in the subsequent interference removal step cannot be adjusted to acidity, resulting in the inability of protein precipitation and recovery. Therefore, 50 mg/mL Reb M in 1 M NaOH was established as the optimal condition for subsequent analyses.

**TABLE 2:** Protein recovery from Reb M with different NaOH concentrations.

Solvent	Reb M /(mg/mL)	Spiked BSA amount /(mg/L)	Measured BSA amount /(mg/L)	Recovery rate /%
0.5 M·NaOH	50	20	17.74	88.70
1 M·NaOH	50	20	19.65	98.25
2 M·NaOH	50	20	0	0.00

In our experiment, we measure the protein content by measuring the absorbance at 562 nm via the BCA method. It is essential to design the interference removal method to reduce the absorbance of Reb M itself. The interference removal protocol is described in Section 2.4. Meantime, to assess the anti‐interference effect of the pretreatment process on Reb M, we prepared a sample solution from the Reb M‐2301 batch following the method outlined in Section 2.3. Different numbers of pretreatment steps were performed to evaluate the impact of varying processing times on interference reduction. As shown in Table 3, the absorbance of the Reb M solution was 0.183 using the unmodified BCA method, which was higher than that of the 7.5 mg/L BSA solution, indicating significant interference with the BCA detection reagents at a concentration of 50 mg/mL. After the first pretreatment step, the background interference absorbance of Reb M decreased from 0.183 to 0.021, a reduction of 88%. Upon repeating the pretreatment process, the absorbance of the solution was effectively reduced to near zero, indicating complete elimination of background interference. These results demonstrate that the modified BCA method with dual pretreatment cycles is required to fully mitigate interference in the BCA assay, ensuring accurate protein quantification.

**TABLE 3:** Effect of pretreatment on the background absorbance of Reb M.

Sample	Processing mode	Absorbance/(AU)
50 mg/mL Reb M	Unmodified BCA method	0.183
50 mg/mL Reb M	Modified BCA method (first pretreatment)	0.021
50 mg/mL Reb M	Modified BCA method (second pretreatment)	0.001
7.5 mg/L BSA	Unmodified BCA method	0.168

### 3.2. Method Validation

#### 3.2.1. Linearity, LOD, and LOQ

**FIGURE 1:**
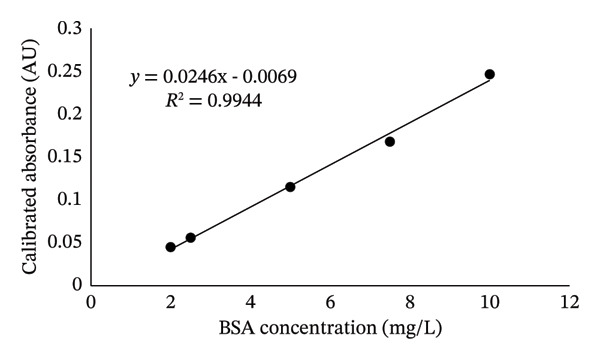
The calibration curve of protein BSA.

The linearity of the BSA solution after pretreatment was evaluated according to the guideline in Section 2.6. The BSA reference solution, prepared as outlined in Section 2.3, was measured using sodium hydroxide solution as the blank control, following the method described in Section 2.4. The absorbance of the blank was subtracted from the measured absorbance to obtain the calibrated absorbance. A standard curve was plotted with the calibrated absorbance and the BSA reference solution concentration at five concentrations ranging from 2 to 10 mg/L in triplicate. The resulting equation shown in Figure 1 was *y* = 0.0246*x*‐0.0069, with a slope (*k*) of 0.0246. The correlation coefficient (*r*) for this range was found as high as 0.99, confirming the robust linearity of the method for pretreated BSA quantification. As summarized in Table 4, the LOD for this method was approximately 0.61 mg/L, which is equivalent to 12.2 mg/kg based on a Reb M and Reb D solution concentration of 50 mg/mL. However, the detection limit for Reb A is consistent with the EU detection limit of 5 mg/kg based on a Reb A solution concentration of 120 mg/mL. Similarly, the LOQ was calculated to be approximately 1.86 mg/L. As per methodological validation requirements, the LOQ of 1.86 mg/L was confirmed for accuracy and precision. This LOQ corresponds to 40 mg/kg (w/w), calculated based on a 50 mg/mL Reb M and Reb D solution concentration. The LOQ is 16 mg/kg for the Reb A. Given that the typical protein residue limit for food additives is around 100 mg/kg, the LOQ of this method is significantly lower than the established limit, ensuring that it can accurately quantify protein residues in Reb M products within the 100 mg/kg threshold.

**TABLE 4:** Methodological detection and quantification limits of Reb M protein residues.

No.	Absorbance /AU	Standard deviation/%	Limit of detection /(mg/L)	Limit of quantitation /(mg/L)
1	0.195	0.458	0.61	1.86
2	0.201			
3	0.204			

#### 3.2.2. Accuracy and Precision

The recovery rate of BSA in both Reb M and Reb D solutions was evaluated at three different spiked concentrations: low (40 mg/kg), medium (100 mg/kg), and high (200 mg/kg), prepared from batch Reb M‐2301 and Reb D‐230701, as described in Sections 2.3 and 2.4. The results are presented in Tables 5 and 6. The recovery rates for BSA across all spiked concentrations ranged from 95.7% to 109.40%, with the SD and repeatability RSD values below 5% (*n* = 3). These results demonstrate that the method meets the quantitative detection requirements for residual proteins in Reb M matrices. The recovery rate of BSA in the Reb A solution was evaluated at three different spiked concentrations: low (16 mg/kg), medium (41 mg/kg), and high (83 mg/kg), prepared from batch Reb A‐230506. The results are shown in Table 7. Under three levels of standard addition, the recovery rate of BSA is between 90.6% and 96.1%, with SD and precision RSD not exceeding 5%, meeting the quantitative detection requirements for residual proteins in Reb D.

**TABLE 5:** Reb M protein residue methodological accuracy and precision.

No.	Spiked amount /(mg/L)	Measured amount /(mg/L)	Recovery rate/%	Standard deviation/%	Average recovery rate/%	RSD /%
1	2	2.09	104.4	3.4	104.5	3.3
	2	2.16	108.0			
	2	2.02	101.2			

2	5	5.30	106.0	2.9	106.3	2.7
	5	5.47	109.4			
	5	5.18	103.6			

3	10	10.09	100.9	0.6	101.5	0.5
	10	10.15	101.5			
	10	10.20	102.0			

**TABLE 6:** Reb D protein residue methodological accuracy and precision.

No.	Spiked amount)/(mg/L)	Measured amount/(mg/L)	Recovery rate/%	Standard deviation/%	Average recovery rate/%	RSD/%
1	2	1.91	95.7%	5.0	101.5%	5.0
	2	2.09	104.4%			
	2	2.09	104.4%			

2	5	5.08	101.6%	2.3	100.3%	2.3
	5	4.88	97.6%			
	5	5.08	101.6%			

3	10	9.79	97.9%	1.4	99.6%	1.4
	10	10.04	100.4%			
	10	10.04	100.4%			

**TABLE 7:** Reb A protein residue methodological accuracy and precision.

No.	Spiked amount)/(mg/L)	Measured amount/(mg/L)	Recovery rate/%	Standard deviation/%	Average recovery rate/%	RSD/%
1	2	1.79	89.4%	3.7	90.6%	4.2
	2	1.81	90.5%			
	2	1.84	91.9%			

2	5	4.56	91.1%	3.8	91.1%	4.2
	5	4.37	87.3%			
	5	4.75	94.9%			

3	10	9.61	96.1%	1.5	94.4%	1.6
	10	9.38	93.8%			
	10	9.33	93.3%			

Meanwhile, the interday reproducibility was also examined. Prepare a 100% concentration Reb M solution and conduct daily parallel testing on 6 samples. The results are shown in Table 8, with an average daytime value of 5.20 mg/L, a SD of 0.16%, and a RSD of 3.1%, indicating good intermediate precision of this method.

**TABLE 8:** Reb M protein residue methodological intermediate precision.

No.	Measured amount)/(mg/L)	Average within group)/(mg/L)	Standard deviation/%	RSD/%	Mean value between groups)/(mg/L)	Intergroup standard deviation/%	RSD/%
Day 1	5.04	5.14	0.14	2.6			
	5.12			
	5.16			
	5.04			
	5.40			
	5.12			

Day 2	5.22	5.33	0.18	3.3	5.20	0.16	3.1
	5.10						
	5.26						
	5.46						
	5.38						
	5.59						

Day 3	5.22	5.13	0.08	1.6			
	5.14			
	5.14			
	5.22			
	5.06			
	5.02			

### 3.3. Sample Analysis

The protein residue levels of Reb M samples from different process batches and final product batches were examined. Reb M‐Process 1 and Reb M‐Process 2 represent crude products from different stages of the production process, while Reb M‐2301, Reb M‐2302, and Reb M‐2303 are the final products. Residual protein levels in the samples were determined according to the method outlined in Section 2.4. As shown in Table 9, protein residues were detectable in the crude products from the Reb M‐process, with significant variation in the amount of residual protein. Notably, the protein residue in the Reb M‐Process 2 batch was already below 100 mg/kg. In contrast, the final product batches showed protein levels below the LOQ of the method, indicating that the final processing stages effectively reduced protein residues in Reb M to levels below 40 mg/kg. These findings confirm that the protocol successfully quantifies protein contamination across a range of sample types, from crude intermediates to purified final products, validating its robustness for quality control throughout the Reb M manufacturing workflow. This analytical capability ensures reliable monitoring of process efficiency in eliminating proteinaceous impurities.

**TABLE 9:** Reb M sample test results.

No.	Batch number	Reb M concentration /(mg/mL)	Measured amount /(mg/L)	Residual protein /(mg/kg)	Standard deviation/%
1		49.21	7.55	153.35	0.40
	Reb M‐Process 1	48.68	7.46	153.20	
		49.96	7.62	152.60	

2		49.62	4.37	88.07	3.74
	Reb M‐Process 2	50.01	4.05	80.98	
		49.83	4.11	82.48	

3		49.85	0.28	5.62(< LOQ)	0.92
	Reb M‐2301	49.64	0.25	5.04(< LOQ)	
		49.81	0.19	3.81(< LOQ)	

4		49.90	0.18	3.61(< LOQ)	0.58
	Reb M‐2302	49.87	0.23	4.61(< LOQ)	
		49.81	0.23	4.62(< LOQ)	

5		49.96	0.26	5.20(< LOQ)	1.10
	Reb M‐2303	49.89	0.15	3.01(< LOQ)	
		50.06	0.20	4.00(< LOQ)	

The modified BCA method was also applied to detect residual proteins in the other three commercially available Reb A, Reb D, and Reb M products. As shown in Table 10, the residual protein in Reb A from Manufacturer 1 ranged from 82.79 to 89.56 mg/kg. Except for Reb M from Manufacturer 4, the Reb D and Reb M products from other manufacturers had residual protein levels exceeding 100 mg/kg. Comparative analysis reveals that Manufacturer 4’s Reb M is the only formulation that meets protein residue compliance, with levels below 40 mg/kg, making it superior to the other Reb A, Reb D, and Reb M products from the other manufacturers.

**TABLE 10:** Commercial stevia sample testing.

No.	Sample	Manufacturers	Batch number	Residual protein /(mg/kg)
1	Reb A	Manufacturer‐1	20230327	82.79
				89.56

2	Reb D	Manufacturer‐2	20240902	109.92
				111.57

3	Reb M	Manufacturer‐3	02BRMGA0122014	121.66
				117.48

4	Reb M	Manufacturer‐4	Z046RP2306	< 40
				< 40

5	Reb M	Manufacturer‐5	240709006	141.39
				139.73

## 4. Conclusions

In this study, we developed and validated a modified BCA method for determining residual proteins in Reb A, Reb D, and Reb M samples, incorporating a protein precipitation pretreatment protocol. A detailed optimization study on the method validation of Reb M was conducted with the most significant variable types of solvent. After comparing the method of Reb A, Reb D, and Reb M, it was found that due to the high solubility of Reb A, the modified BCA method can indeed achieve the detection limit of 5 mg/kg specified by the European Union. However, there is a problem of solubility difference between Reb D and Reb M, and the detection limit can be as low as 12 mg/kg. Compared to traditional BCA methods, this approach effectively addresses the matrix interference common in Reb M samples, offering notable advantages in analyzing complex and interference‐prone matrices. The results demonstrated that trace amounts of BSA could be efficiently recovered after pretreatment, with satisfactory detection sensitivity and accuracy. The method showed excellent linearity, with correlation coefficient (*r*) values exceeding 0.99. This method is currently with the lowest detection limit publicly disclosed and has been fully validated. In conclusion, this methodology provides a versatile analytical platform for routine residue protein monitoring in enzyme‐catalyzed products, fulfilling a critical need for contamination control in precision manufacturing processes.

## Funding

This research was funded by the Xi’an Science and Technology Plan (No. 24GXFW0081‐29) and Innovation Training Projects (SC202411080069, DC2024141).

## Disclosure

A preprint has previously been published [Feng Li, Wenjing Zhang, Xinjing Yang, et al. 2025].

## Conflicts of Interest

The authors declare no conflicts of interest.

## Data Availability

The data that support the findings of this study are available from the corresponding author upon reasonable request.
